# Improving adherence to glaucoma medication: a randomised controlled trial of a patient-centred intervention (The Norwich Adherence Glaucoma Study)

**DOI:** 10.1186/1471-2415-14-32

**Published:** 2014-03-24

**Authors:** Heidi Cate, Debi Bhattacharya, Allan Clark, Richard Fordham, Richard Holland, David C Broadway

**Affiliations:** 1 Norfolk & Norwich University Hospital NHS Trust, Glaucoma Research Unit, Level 2, West Block, Colney Lane, Norwich NR4 7UY, UK; 2 School of Pharmacy, University of East Anglia, Norwich NR4 7TJ, UK; 3 Norwich Medical School, University of East Anglia, Norwich NR4 7TJ, UK

## Abstract

**Background:**

Improving adherence to ocular hypertension (OH)/glaucoma therapy is highly likely to prevent or reduce progression of optic nerve damage. The present study used a behaviour change counselling intervention to determine whether education and support was beneficial and cost-effective in improving adherence with glaucoma therapy.

**Methods:**

A randomised controlled trial with a 13-month recruitment and 8-month follow-up period was conducted. Patients with OH/glaucoma attending a glaucoma clinic and starting treatment with travoprost were approached. Participants were randomised into two groups and adherence was measured over 8 months, using an electronic monitoring device (Travalert® dosing aid, TDA). The control group received standard clinical care, and the intervention group received a novel glaucoma education and motivational support package using behaviour change counselling. Cost-effectiveness framework analysis was used to estimate any potential cost benefit of improving adherence.

**Results:**

Two hundred and eight patients were recruited (102 intervention, 106 control). No significant difference in mean adherence over the monitoring period was identified with 77.2% (CI, 73.0, 81.4) for the control group and 74.8% (CI, 69.7, 79.9) for the intervention group (p = 0.47). Similarly, there was no significant difference in percentage intraocular pressure reduction; 27.6% (CI, 23.5, 31.7) for the control group and 25.3% (CI, 21.06, 29.54) for the intervention group (p = 0.45). Participants in the intervention group were more satisfied with information about glaucoma medication with a mean score of 14.47/17 (CI, 13.85, 15.0) compared with control group which was 8.51 (CI, 7.72, 9.30). The mean intervention cost per patient was GB£10.35 (<US$16) and not cost-effective.

**Conclusions:**

Adherence with travoprost was high and not further increased by the intervention. Nevertheless, the study demonstrated that provision of information, tailored to the individual, was inexpensive and able to achieve high patient satisfaction with respect to information about glaucoma medication. Measurement of adherence remains problematic since awareness of study participation may cause a change in participant behaviour.

**Trial registration:**

Current Controlled Trials, ISRCTN89683704.

## Background

Sub-optimal adherence to prescribed glaucoma therapy is associated with progressive visual field loss and unnecessary additional prescribing or surgery [1]. A cross-sectional glaucoma study has found that participants with adherence rates less than 80% have worse visual field defects than those with greater adherence rates [2].

Patients that have a stronger belief in the necessity for eye drops are more adherent [3] and studies that have targeted patient beliefs have been effective in improving adherence [4,5]. Conversely, whilst poor glaucoma education has been cited as an explanation for non-adherence to therapy [6-8], interventions that purely focus on providing education have failed to achieve significant improvement in adherence [9-11]. However, studies using multifaceted intervention components including education and tailoring of information, and encouraging patients to discuss strategies for incorporating the medication administration into their daily activities, have detected a significant improvement in adherence [12,13].

Thus, the aim of the present study was to determine whether an intervention designed to both target beliefs and provide tailored education about glaucoma and its management by using a behaviour change counselling (BCC) technique [14], could be beneficial and cost-effective in improving adherence with topical therapy. The intervention was designed to address the multifactorial nature of unintentional and intentional non-adherence [15]. The design, justification of such an intervention and methodology of outcome measures is the subject of a previous published protocol paper by the same authors [16]. Previous research with the same patient population highlighted that an intervention that provided glaucoma and treatment information and motivational support was required at the point of medication initiation [7]. Therefore, paramount to the design of the intervention was the feasibility of the randomisation and delivery of the intervention during the same clinic visit as initiation of treatment and an 8 month follow-up period to establish the longevity of any intervention effect on adherence.

## Methods

### Sample and setting

The Norwich Adherence Glaucoma Study (NAGS) was a randomised controlled trial conducted in the Glaucoma Clinic of a UK National Health Service (NHS) teaching hospital. The study received ethical approval from the Norfolk Research Ethics Committee, UK. A summary of the methods are provided since the full details have been described previously [16].

### Patient recruitment, randomisation and blinding

Consecutive patients newly initiated onto travoprost monotherapy for the treatment of primary open angle glaucoma (POAG), ocular hypertension (OH) or glaucoma suspect using the European Glaucoma Society Guidelines [17], were invited to participate; no financial stipend or travoprost samples were given, and patients collected their own repeat prescription for travoprost as per standard UK practice. Patients interested in study participation were referred by their clinician to the research staff and were informed that they may be randomised to either the standard care or the intervention group (allocation ratio 1:1), and that their adherence to travoprost would be monitored for the study duration. No standardized script was used during recruitment.

Participants giving written consent were randomised, stratified by diagnosis (either POAG, or OH/glaucoma suspect) and experience of the glaucoma service (new patient or follow-up patient but commencing active treatment) by research staff using a centralized automated telephone randomisation system. Given the interventional nature of the study, patients and research staff were not masked to their intervention allocation, but clinicians were masked as to which patients were participating in the study. The statistician (AC) was also masked to which data belonged to the intervention/control group during analysis. Once randomised, all participants were followed through to study completion regardless of changes to therapy, including travoprost discontinuation.

Basic demographic information were collected for non-consenting patients.

### Study design

Five nurses/research technicians working within the glaucoma clinic were trained to deliver the intervention and study related tasks as previously described [16]; they were titled ‘Glaucoma Support Assistants (GSAs)’ during the study. Two of the five GSAs were Glaucoma Specialist Nurses delivering routine nurse-led glaucoma patient review clinics. The GSA specialist nurses separated split roles by delivering routine care three days a week and undertaking the GSA role on one separate day a week. To prevent the roles of GSAs influencing the provision of routine care, administrative staff ensured that participants did not attend nurse-led follow-up glaucoma clinics run by nurses with split roles. Furthermore, pre-randomisation, patients were unlikely to have received care from the GSA nurses as Glaucoma Specialist Nurses do not prescribe medication and would not have been exposed to patients newly requiring treatment.

### The control

Standard care was provided which involved appropriate tests to establish POAG/OH diagnosis and initiation of treatment if indicated before entering the study. All participants received basic information and leaflets about glaucoma as deemed appropriate by their clinicians, who were not associated with the research. Follow-up was in accordance with standard clinical protocols.

### The intervention

Before leaving the Glaucoma Clinic, intervention participants received an individualised glaucoma education and motivational support package using BCC from a GSA. The development and standardisation of the intervention has been previously described [16]. A telephone advice-line from Monday to Friday, 9 am–5 pm was provided by the GSAs to respond to glaucoma related queries from participants and their carers.

## Data sources: adherence monitoring, questionnaires, medical records and interviews

### Travalert® dosing aid (TDA)

The Travalert® dosing aid (TDA, Alcon Laboratories, Inc., Forth Worth, TX, USA) was used to collect adherence data from all participants. Each participant had their adherence calculated using a computer programme specifically designed for the purpose. The Steering Committee for the study, which included glaucoma specialist clinicians, adherence experts, a patient using eye drops for glaucoma and public representative, discussed and agreed what would determine an adherent dose; at least one recorded application (regardless of bi- or uni-lateral dosing) within a 4-hour window either side of an individual’s average dosing time; the mean of each daily dose administered between 5 pm and 5 am was calculated for the duration of the study period. Eye drop application using the TDA was demonstrated to all participants by a GSA, irrespective of group allocation. New TDAs were dispensed at visit-2 (2 months) if the GSA found the battery or TDA to have failed during patient use.

### Questionnaires

**Figure 1:**
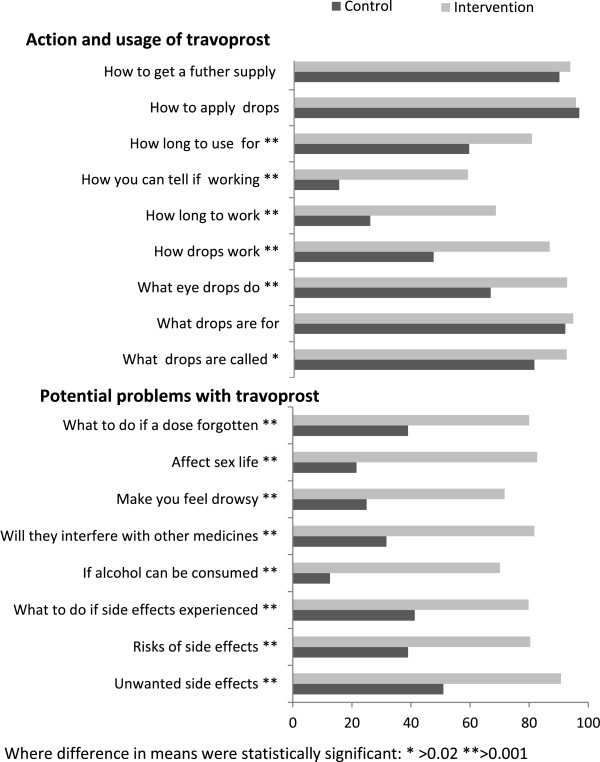
Comparison of the control and intervention group SIMS at visit 1 categorised according to items that relate to potential problems with travoprost and action and usage of travoprost.

A participant self-administered questionnaire was completed after visits-1 (study initiation), 2 (2 months) and 3 (8 months) and returned by post. The questionnaire included self-reported satisfaction with information received about Travoprost using the validated ‘Satisfaction with Information about Medicines Scale’ (SIMS) [18]. SIMS comprises 17 questions which are detailed in Figure 1 and scores range from 0 to 17 with high scores indicating greater satisfaction.

### Medical records

Glaucoma Clinic held records were accessed for IOP measured by Goldmann tonometry at baseline, and visit-3 (8 months) plus co-morbidity data which was classified using the Charlson Co-morbidity Index [19].

### Interviews

A structured interview undertaken at the time of recruitment was used to collect patient age, ethnicity (classified by participants), index of multiple deprivation (IMD) (a UK specific measure of relative deprivation using postal addresses), type of housing, educational qualifications and family history of glaucoma; these characteristics were used to describe the participant population and explore predictors of adherence (not reported in this publication).

### Primary outcomes

Adherence was determined using the number of adherent doses using electronic monitoring divided by the expected number of doses for the monitoring period for both the total 8 month study duration and the final 2 months of follow-up [16]. Additionally, a dichotomous classification of adherence score based on the final 2 months of follow-up was used (‘adherent’ if the average number of TDA recorded doses was ≥80% of expected and ‘non-adherent’ if <80%).

### Secondary outcomes

The IOP measure for each treated eye was used as a clinical outcome measure. The percentage reduction in IOP from baseline and visit-3 was calculated and compared between intervention and control groups.

The SIMS score was used to establish differences between the control and intervention groups’ satisfaction with information received about their medication. The SIMS score was also used compared with participants adherence score to ascertain if satisfaction with information improved adherence to medication.

### Health economic measures

A resource log book captured all Glaucoma Clinic care activity and GSA time spent with each participant delivering the education and support package, in addition to answering any queries on the telephone helpline. Patient costs were excluded from the study. The health economic analysis was based upon the NHS for England perspective. The primary outcome measure was incremental hospital cost per percentage gain in adherence. The specific costs associated with secondary care ophthalmic activity were identified on review of the patient pathway by an expert clinician (DCB), glaucoma health care professional (HC), and health economist (RF). The costs captured in the resource logs were described using direct hospital treatment costs [16].

## Missing data

Missing data were imputed using a multivariate normal imputation model after suitable transformations, to ensure that the variables were normally distributed. A sensitivity analysis was carried out to assess the effects of missing data. The results (not shown) indicated that results were not sensitive to the missing data.

## Therapist bias

Since the intervention was intended to improve adherence by motivating patient behaviour, all GSA interventions were examined to test for any potential ‘therapist bias’. Interventions were grouped by the GSA delivering the intervention and the number of adherent participants per GSA compared using a Chi-squared test. Any GSA showing greater or lesser adherence outcome per participant, may have suggested a potential significant bias in the way that an individual GSA had delivered the intervention to their allocated participants.

## Statistical analysis

Descriptive statistics were used to characterise the demographics of the study population. All analyses were based on an intention-to-treat principle.

The primary analysis compared the percentage of intervention and control group ‘adherers’ using a Chi-squared test for the total 8 months of follow-up and separately for the final 2 months. Additionally, the combined month-7 and −8 post-randomisation percentage adherence values were analysed using a Student’s t-test to compare the intervention and control group. A repeated measures analysis-of-variance (ANOVA) was carried out (with time measured in months) to assess for any difference between intervention and control over time.

Reduction in IOP, and SIMS were compared between the control and intervention groups using Student’s t-tests and at different time points (SIMS and IOP at visit-1, visit-2 and visit-3) to compare any differences. Adherence was correlated with SIMS using a Spearman’s rank correlation coefficient (SRCC) test.

The difference in total costs and adherence percentage was calculated incrementally between the intervention and control group to provide the incremental cost effectiveness ratio (ICER) of additional adherence from the intervention. A deterministic sensitivity analysis was performed on the base case ICER using patient variability in cost and outcome to determine the robustness of the economic analysis.

## Sample size

At the time of calculating the sample size, there was less available data to suggest likely adherence rates with once daily glaucoma medication [20] and limited research to indicate the effect of an intervention on adherence. Thus sample size estimates were derived from general medicine where average non-adherence rates of 25% have been reported [21,22]. Available data at the time for glaucoma adherence studies using medication monitors had shorter follow-up time and more complicated dosing regimens than the proposed study [23,24]. Therefore, without a comparable study, a 20% increase in adherence was estimated and adherence defined as ≥ 80% of expected doses recorded by the TDA. Assuming an adherence rate of 60% in the control group and 80% in the intervention group, 81 people in each group provided 80% power to detect a difference using a Chi-squared test with 5% significance. Based on an estimated 20% drop-out a target of 200 participants was set.

## Results

**Figure 2:**
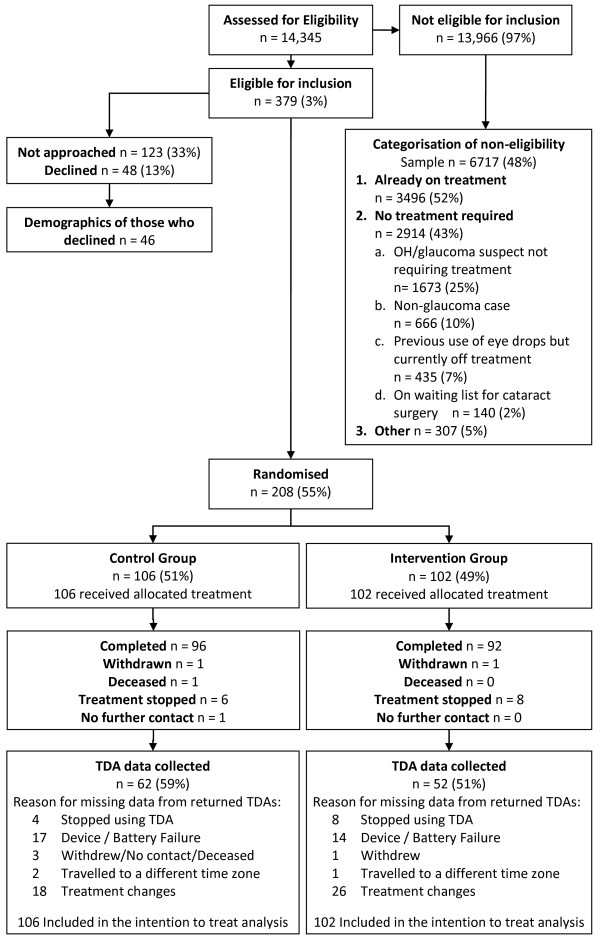
The Consolidated Standards of Reporting Trials diagram including detailed information on excluded participants and missing TDA data.

Eligible participants were recruited from November 2009 for 13 months as illustrated in Figure 2. Previous use of glaucoma therapy was the most frequent reason for exclusion. There was no suggestion that the failure of the TDA device differed between those who were adherent and those who were not. Furthermore, participants who changed treatment were not associated with poorer adherence.

The demographic characteristics of the sample population are summarised in Table 1 and were evenly balanced. The participants were predominantly of white British ethnicity and, at the time of recruitment, had been diagnosed with POAG as opposed to OH or glaucoma suspect. The cohort was evenly matched as to whether they were new patients or had been seen previously in the Glaucoma Clinic. There were no statistically significant differences in age (p = 0.257), gender (p = 0.253) and IMD (p = 0.379) between study participants and the 46 individuals who declined participation.

**Table 1:** Baseline characteristics for control and intervention groups

	**Control**		**Intervention**	
	**N**	**%**	**N**	**%**
Gender				
Male	58	54.7	47	46.1
Female	48	45.3	55	53.9
Ethnicity				
White British	104	98.1	102	100
Other	2	1.9	0	0
Housing tenure				
Home owner	92	86.8	82	80.4
Renter (council)	6	5.7	11	10.8
Renter (private)	5	4.7	4	3.9
Other	3	2.8	5	4.9
Marital status				
Married/partner	75	70.8	73	71.6
Divorced/separated	5	4.7	7	6.9
Widowed	19	17.9	17	16.7
Single	7	6.6	5	4.9
Highest qualification				
GCSE (high school)	44	41.5	45	44.1
A-levels (college)	4	3.8	5	4.9
Degree (graduate school)	11	10.4	8	7.8
Post-graduate	8	7.5	2	2
Other (inc. less than GCSE)	37	34.9	42	41.2
Parents with glaucoma				
No	79	74.5	70	68.6
Yes	22	20.08	24	23.5
Not known/no contact	5	4.7	8	7.8
Siblings with glaucoma				
No	93	87.7	85	83.3
Yes	8	7.5	9	8.8
Not known/no contact	5	4.7	8	7.8
Children with glaucoma				
No	102	96.2	95	93.1
Yes	2	1.9	0	0
Not known/no contact	2	1.9	7	6.9
Diagnosis and new/follow-up care				
POAG/NTG new patient	33	31.1	32	31.4
POAG/NTG follow-up patient	40	37.7	37	36.3
GS/OH new patient	16	15.1	15	14.7
GS/OH follow-up patient	17	16.0	18	17.6
	**Mean**	**SD**	**Mean**	**SD**
Age	70.06	10.9	70.7	11.3
Intraocular pressure	23.4	10.9	22.2	5.4
Charlson score	1.6	2.2	1.5	2.1
Number of medications	2.8	2.9	2.7	2.8
IMD	12.7	7.7	15.4	11.2

There were no significant differences between observed and imputed data, thus all analyses were conducted on observed data and there were no therapist effects (Chi-Squared p = 0.860).

**Figure 3:**
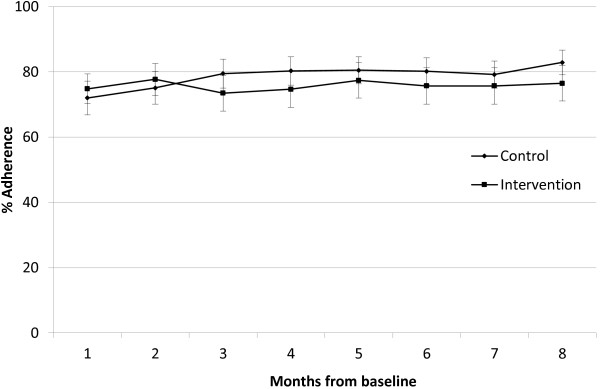
Monthly mean % adherence (CI) for control and intervention groups.

Mean adherence over the total 8-month monitoring period was slightly greater in the control group (77.2%), but the difference (2.4%; 95% CI, −4.2, 9.0) between the two groups was small and not statistically significant (p = 0.471). There was also no difference in the mean adherence for the final 2 months of monitoring which was 79.3% in the control group, the difference (1.6%; 95% CI, −6.8, 10.0) between the two groups being minimal (p = 0.703). There was no statistically significant difference in the proportion of individuals with ≥ 80% adherence (p = 0.631) between the two groups: control group 62.5% and intervention group 66.7%. A repeated measures analysis of percentage adherence rate for each month found no difference in adherence between the two groups (p = 0.685) or any interaction between month and group (p = 0.894), the details of which are provided in Figure 3.

### Intraocular pressure

The mean IOP at baseline for subsequently treated right and/or left eyes was 23.7 mmHg (95% CI, 22.6, 24.8) for the control group and 22.4 mmHg (95% CI, 21.3, 23.5) for the intervention group which was not statistically different (p = 0.096, 1.3, CI −0.2, 2.9). There was no significant difference in percentage reduction in IOP from baseline to visit-3 between the two groups with control group being 27.6% (SD, 19.2) and intervention group being 25.3% (SD 19.7) (2.3 mean difference, CI −3.6, 8.2, p = 0.448).

### Satisfaction with information about travoprost

SIMS scores for the intervention group were significantly higher than the control group at all three time points post intervention (visit-1 mean score = 14.47 (95% CI, 13.85, 15.09) for the intervention group and 8.51 for the control group (95% CI, 7.72, 9.30, p < 0.001). The same high level of satisfaction was maintained for the intervention group, whilst control group SIMS score did increased significantly over time with a difference in mean score between visit-1 and visit-3 of 2.91, SD 4.74 p = <0.001. SIMS was not significantly related to medication adherence (r = −0.015 p = 0.883).

Figure 1 provides a comparative illustration of the specific items of SIMS information that intervention and control participants felt they lacked and these were categorised into items related to ‘action and usage’ or ‘potential problems of travoprost’ [18]. Both groups had a higher degree of satisfaction with information about ‘action and usage of travoprost’ (control group mean average 65%, intervention group 86%) than the ‘potential problems’ (control group 33%, intervention group 80%) but the difference was considerably more marked in the control group.

### Health economic evaluation

The mean intervention cost per participant was GB£10.35 with a total expenditure GB£746.17 greater than the control group. Since no significantly greater effectiveness was found (percentage gain in adherence) but it was delivered at a higher cost, the intervention was not cost-effective.

## Discussion

The present trial of an adherence intervention failed to achieve greater adherence to newly initiated topical glaucoma treatment. Adherence in the control group was considerably higher than the previously reported estimates used in general medicine, that were used in the present study power calculations [22] and ophthalmology [25]. Thus, improving adherence, in an already adherent population, would have been difficult to achieve.

Previous studies examining interventions to improve adherence to glaucoma medication have enrolled patients identified to be poorly adherent in an attempt to create the best conditions to measure greater effect sizes [26] or have measured adherence pre- and post- intervention to make comparison of individual differences [13]. However, the present study examined the potential of an intervention to improve adherence at the point of medication initiation in accordance with previous research findings [7]. Since patients were medication naïve, targeting unintentionally non-adherent patients was not feasible. Furthermore, clinical practice at the time of the study could not have accurately predicted patients likely to have poor adherence [27] and measuring such behaviour would have required several months to perform and these observational methods often alert patients to the fact that their behaviour is being monitored which can cause a reactivity bias resulting in increased adherence to medication [28,29]. Thus, it was neither appropriate nor feasible to target a poorly adherent cohort in the present study.

The present study result may indicate that standard care offered by an NHS Glaucoma Clinic was sufficient to promote high adherence with travoprost for the population studied. An alternative interpretation of the high level of adherence is that Hawthorne effects may have occurred, whereby the act of study participation improved motivation and thus increased adherence in both groups [26,28,30]. Future studies may benefit from including a placebo ‘attention’ arm in order to control for the extra attention that participants receive when they agree to take part in research. Standardisation of instructions given to participants at the time of recruitment may also control for external variances in measured adherence caused by the way information is conveyed and comprehended. However, these phenomena are not well documented and further research is required to establish the extent that study participation and reactivity to adherence monitoring may inflate adherence relative to the natural environment. Furthermore, studies of adherence may be intrinsically biased through selection of patients who attend appointments and engage in healthcare; thus non-adherent patients are more likely to be missing from the sample and those agreeing to participate may be more adherent to medication than those who decline [31].

Another consideration is that the fidelity of intervention delivery may have been compromised. However, as described in the protocol paper [16], a rigorous training process for GSAs was undertaken and quality assured by 3 experts in motivational interviewing.

Finally, the intervention may have been flawed in its design. Since conception of the intervention used in the present study, the large body of evidence to identify the domains that explain behaviour change has been collated into the Theoretical Domains Framework [32,33]. The present intervention design which was informed by empirical evidence as described in the protocol paper [16] maps comprehensively onto the Theoretical Domains Framework [34]. However, given that the participants were treatment naïve, after using their eye drops in daily life, their underlying behavioural processes may have altered. For example, the domain called beliefs about capabilities, is particularly susceptible to change after the participant has experienced using their eye drops. Whilst the participant may have felt empowered and capable within the clinic environment, the home environment may present new unanticipated challenges. It may therefore be appropriate for a subsequent study to include a second consultation perhaps targeted at non-adherent participants.

In so far as the authors know, the present study is the first that has attempted to report 8-months of follow-up using a TDA. Previous studies have reported that the TDA accurately records drop administration, but the longest of these studies was for only a 3-month duration [35-37]. At month 2 only 10% of the TDA was missing, which suggests it was the longevity of the present study that lead to the high failure of the TDA. Whilst the failure of the TDA increased the uncertainty around the adherence estimate, there was no evidence that the failure of the TDA differed between those who were adherent and those who were not, and thus did not bias the results.

Reduction of IOP did not correlate with TDA measured adherence, most likely due to limitations of the methodological approach and the pharmacodynamics of prostaglandin analogues, rather than conclusive evidence that the intervention failed to improve IOP control. However, previous studies using similar methods have also found that IOP reduction had no relationship to adherence [13,37]. Assessing IOP due to individual differences (types of glaucoma and diurnal variance) together with regression to the mean, led to ‘noisy data’ [38,39]. Better measures of both adherence and clinical outcomes are needed for future glaucoma research, but what these might be, remains difficult to establish.

Contrary to evidence from previous studies, whilst the NAGS intervention group were more satisfied with information received about travoprost, this had no measurable effect on adherence which may be due to any number of the reasons already discussed. Since the most marked difference between intervention and control groups related to potential problems of using travoprost, standard care would appear to require greater information provision with respect to these aspects, but whether this would improve adherence has not been established. The increase over time in information satisfaction demonstrated by the control group is interesting and suggests that patients seek/obtain information from additional sources post treatment initiation or that the desire for information declines.

The NAGS cost effectiveness analysis was based upon improvement in adherence between the two groups and as such the data were dominated by the control treatment. Utilising a quality-adjusted life-years (QALY) analysis to measure the impact of the intervention on health may have provided useful additional information. However, generic preference instruments such as the Sickness Impact Profile and Short Form Health Survey-6D were not considered specific enough to detect the asymptomatic nature of glaucoma in its early stages. Furthermore, it is unlikely that glaucoma specific tools would detect patient quality of life changes associated with early stages of disease in an 8 month period of follow-up, since in the majority of treated and monitored patients, disease progression is slow [40-42].

There is little published evidence to suggest that adherence interventions can consistently improve adherence to medication within the resources available in clinical settings. Interventions are commonly led by research teams which cannot easily be translated into clinical practice [22]. However, in this study the intervention was led by specialist nurses and allied health professionals already working within the hospital eye service. The economic costs associated with the provision of the intervention were also based upon local costs, which provided a more realistic consideration of associated costs. Whilst this gave confidence in the ease as to how such an intervention could be incorporated into clinical practice and its relative cost, ensuring that there was no contamination between allied health professionals who worked in the Eye Clinic (and had received enhanced training in motivational interviewing skills), was more problematic. Every effort was made to ensure that participants did not come into contact with GSAs during their follow-up period, but it was not possible to account for any influences these staff and the study itself had on routine practices of all clinical staff during the study period.

The results of the present study relate to a relatively affluent, primarily white British population prescribed glaucoma mono-therapy, and may not be generalisable to other populations that may have different cultural practices. Mono-therapy is also thought to aid adherence, with more complex regimens being problematic for patients, [7,43] so the results should not be extrapolated to patients prescribed dual or multiple therapies. It would be of value to study the effect of the present intervention on a population expected to have lower adherence and using multiple topical anti-glaucoma therapies.

Whilst a standardised measure for adherence remains undefined, studies continue to produce heterogeneous adherence results which may be due to variations in adherence measurement, duration of monitoring and definition of adherence. In the present study, we used multiple measures and reporting methods of adherence which provided consistent findings and ensured that comparisons could be made with studies of shorter duration and those using differing statistical analyses.

## Conclusion

In conclusion, the present study did not demonstrate improved adherence with a behaviour change intervention, despite the intervention producing high levels of patient satisfaction. However, whilst previous negative results have often been under-reported in favour of studies with significant findings [44] publication of this study, which was informed by theory plus empirical evidence and adhering to rigorous study methods, is therefore of significance to researchers and health professionals in this field. The model described in the present study requires further development as a potential service to give better support to patients prescribed topical glaucoma therapy. However, measurement of adherence itself and determination of the long-term clinical benefits of improving adherence remain problematic. In particular, further research is required to determine the size of any Hawthorne effects. Whilst a standardised and accurate measure for adherence remains undefined, future adherence studies may benefit from utilising multiple methods to quantify and classify adherence in parallel to help clarify true adherence levels.

## Competing interests

The authors declare that they have no competing interests.

## Authors’ contributions

HC, DB, DCB, AC, RF, RH conceived of the study, and participated in its design and coordination and helped to draft the manuscript. AC also performed the statistical analysis. RF also advised on the health economic analysis. All authors read and approved the final manuscript.

## Pre-publication history

The pre-publication history for this paper can be accessed here:

http://www.biomedcentral.com/1471-2415/14/32/prepub

## References

[B1] Denis P Lafuma A Berdeaux G Medical predictive factors of glaucoma treatment costs J Glaucoma 2004 13 4 283 290 10.1097/00061198-200408000-00005 15226656

[B2] Sleath B Blalock S Covert D Stone JL Skinner AC Muir K Robin AL The relationship between glaucoma medication adherence, eye drop technique, and visual field defect severity Ophthalmology 2011 118 12 2398 2402 10.1016/j.ophtha.2011.05.013 21856009 PMC3223548

[B3] Stryker JE Beck AD Primo SA Echt KV Bundy L Pretorius GC Glanz K An exploratory study of factors influencing glaucoma treatment adherence J Glaucoma 2010 19 1 66 72 10.1097/IJG.0b013e31819c4679 20075676 PMC2808197

[B4] Kripalani S Yao X Haynes RB Interventions to enhance medication adherence in chronic medical conditions: a systematic review Arch Intern Med 2007 167 6 540 550 10.1001/archinte.167.6.540 17389285

[B5] Gray TA Fenerty C Harper R Spencer AF Campbell M Henson DB Waterman H Individualised patient care as an adjunct to standard care for promoting adherence to ocular hypotensive therapy: an exploratory randomised controlled trial Eye (Lond) 2012 26 3 407 417 10.1038/eye.2011.269 22094303 PMC3299012

[B6] Gray TA Fenerty C Harper R Lee A Spencer AF Campbell M Henson DB Waterman H Preliminary survey of educational support for patients prescribed ocular hypotensive therapy Eye (Lond) 2010 24 12 1777 1786 10.1038/eye.2010.121 20829888

[B7] Lacey J Cate H Broadway DC Barriers to adherence with glaucoma medications: a qualitative research study Eye (Lond) 2009 23 4 924 932 10.1038/eye.2008.103 18437182

[B8] Taylor SA Galbraith SM Mills RP Causes of non-compliance with drug regimens in glaucoma patients: a qualitative study J Ocul Pharmacol Ther 2002 18 5 401 409 10.1089/10807680260362687 12419091

[B9] Domino FJ Improving Adherence to Treatment for Hypertension Am Fam Physician 2005 71 11 2089 2090 15952435

[B10] Beckers HJ Webers CA Busch MJ Brink HM Colen TP Schouten JS Adherence improvement in Dutch glaucoma patients: a randomised controlled trial [published online ahead of print October 1 2012] Acta Ophthalmol 2013 91 7 610 618 10.1111/j.1755-3768.2012.02571.x 23025424

[B11] Sheppard J Waner J Kelley K An evaluation of the effectiveness of a nurse-led glaucoma montiroring clinic Ophthalmic Nurs 2003 7 2 15 21

[B12] Norell SE Improving medication compliance: a randomised clinical trial Br Med J 1979 2 6197 1031 1033 10.1136/bmj.2.6197.1031 519269 PMC1596809

[B13] Okeke CO Quigley HA Jampel HD Ying GS Plyler RJ Jiang Y Friedman DS Interventions improve poor adherence with once daily glaucoma medications in electronically monitored patients Ophthalmology 2009 116 12 2286 2293 10.1016/j.ophtha.2009.05.026 19815286 PMC2787727

[B14] Rollnick S Allison J Ballasiotes S Barth T Butler C Rose G Rosengren D Miller WR, Rollnick S Variations on a theme: motivational interviewing and its adaptations Motivational Interviewing: Preparing People for Change 2002 New York: Guildford Press 270 283

[B15] Haynes RB Haynes R, Taylor D, Sakett D Determinants of compliance: the disease and the mechanics of treatment Compliance in Health Care 1979 Baltimore, USA: The Johns Hopkins University Press 49 62

[B16] Cate H Bhattacharya D Clark A Fordham R Notley C Broadway DC Protocol for a randomised controlled trial to estimate the effects and costs of a patient centred educational intervention in glaucoma management BMC Ophthalmol 2012 12 1 57 10.1186/1471-2415-12-57 23171166 PMC3536708

[B17] European Glaucoma Society Guidelines http://www.eugs.org/eng/EGS_guidelines.asp

[B18] Horne R Hankins M Jenkins R The Satisfaction with Information about Medicines Scale (SIMS): a new measurement tool for audit and research Qual Health Care 2001 10 3 135 140 10.1136/qhc.0100135 11533420 PMC1743429

[B19] Charlson ME Pompei P Ales KL MacKenzie CR A new method of classifying prognostic comorbidity in longitudinal studies: development and validation J Chronic Dis 1987 40 5 373 383 10.1016/0021-9681(87)90171-8 3558716

[B20] Olthoff CM Schouten JS van de Borne BW Webers CA Noncompliance with ocular hypotensive treatment in patients with glaucoma or ocular hypertension an evidence-based review Ophthalmology 2005 112 6 953 961 10.1016/j.ophtha.2004.12.035 15885795

[B21] Schwartz GF Compliance and persistency in glaucoma follow-up treatment Curr Opin Ophthalmol 2005 16 2 114 121 10.1097/01.icu.0000156139.05323.26 15744142

[B22] Haynes RB Yao X Degani A Kripalani S Garg A McDonald HP Interventions to enhance medication adherence Cochrane Database Syst Rev 2005 4 CD000011 16235271 10.1002/14651858.CD000011.pub2

[B23] Kass MA Gordon M Morley RE Jr Meltzer DW Goldberg JJ Compliance with topical timolol treatment Am J Ophthalmol 1987 103 2 188 193 3812621 10.1016/s0002-9394(14)74225-4

[B24] Kass MA Meltzer DW Gordon M Cooper D Goldberg J Compliance with topical pilocarpine treatment Am J Ophthalmol 1986 101 5 515 523 3706455 10.1016/0002-9394(86)90939-6

[B25] Waterman H Evans JR Gray TA Henson D Harper R Interventions for improving adherence to ocular hypotensive therapy Cochrane Database Syst Rev 2013 4 CD006132 23633333 10.1002/14651858.CD006132.pub3 PMC11586094

[B26] Glanz K Beck AD Bundy L Primo S Lynn MJ Cleveland J Wold JA Echt KV Impact of a health communication intervention to improve glaucoma treatment adherence. Results of the interactive study to increase glaucoma adherence to treatment trial Arch Ophthalmol 2012 130 10 1252 1258 22688429 10.1001/archophthalmol.2012.1607 PMC3593648

[B27] Budenz DL A clinician’s guide to the assessment and management of nonadherence in glaucoma Ophthalmology 2009 116 11 Suppl S43 47 19837260 10.1016/j.ophtha.2009.06.022

[B28] Gray TA Orton LC Henson D Harper R Waterman H Interventions for improving adherence to ocular hypotensive therapy Cochrane Database Syst Rev 2009 2 CD006132 19370627 10.1002/14651858.CD006132.pub2

[B29] Litcher-Kelly L Kellerman Q Hanauer SB Stone AA Feasibility and utility of an electronic diary to assess self-report symptoms in patients with inflammatory bowel disease Ann Behav Med 2007 33 2 207 212 10.1007/BF02879902 17447873

[B30] McCambridge J Butor-Bhavsar K Witton J Elbourne D Can research assessments themselves cause bias in behaviour change trials? A systematic review of evidence from solomon 4-group studies PLoS One 2011 6 10 e25223 10.1371/journal.pone.0025223 22039407 PMC3198466

[B31] McDonald HP Garg AX Haynes RB Interventions to enhance patient adherence to medication prescriptions: scientific review Jama 2002 288 22 2868 2879 10.1001/jama.288.22.2868 12472329

[B32] Michie S van Stralen MM West R The behaviour change wheel: a new method for characterising and designing behaviour change interventions Implement Sci 2011 6 42 10.1186/1748-5908-6-42 21513547 PMC3096582

[B33] Michie S Richardson M Johnston M Abraham C Francis J Hardeman W Eccles MP Cane J Wood CE The behavior change technique taxonomy (v1) of 93 hierarchically clustered techniques: building an international consensus for the reporting of behavior change interventions Ann Behav Med 2013 46 1 81 95 10.1007/s12160-013-9486-6 23512568

[B34] Michie S Johnston M Abraham C Lawton R Parker D Walker A Psychological Theory G Making psychological theory useful for implementing evidence based practice: a consensus approach Qual Saf Health Care 2005 14 1 26 33 10.1136/qshc.2004.011155 15692000 PMC1743963

[B35] Okeke CO Quigley HA Jampel HD Ying GS Plyler RJ Jiang Y Friedman DS Adherence with topical glaucoma medication monitored electronically the Travatan Dosing Aid study Ophthalmology 2009 116 2 191 199 10.1016/j.ophtha.2008.09.004 19084273

[B36] Friedman DS Jampel HD Congdon NG Miller R Quigley HA The Travatan Dosing Aid accurately records when drops are taken Am J Ophthalmol 2007 143 4 699 701 10.1016/j.ajo.2006.11.036 17386285

[B37] Ajit RR Fenerty CH Henson DB Patterns and rate of adherence to glaucoma therapy using an electronic dosing aid Eye (Lond) 2010 24 8 1338 1343 10.1038/eye.2010.27 20339390

[B38] Cate H Broadway DC Association between intraocular pressure and adherence: is there one? Eye (Lond) 2011 25 9 1238 1239 21597482 10.1038/eye.2011.111 PMC3178251

[B39] Ajit RR Fenerty CH Henson DB Response to cate and broadway Eye (Lond) 2011 25 9 1238 123921597482

[B40] Kass MA Heuer DK Higginbotham EJ Johnson CA Keltner JL Miller JP Parrish RK 2nd Wilson MR Gordon MO The Ocular Hypertension Treatment Study: a randomised trial determines that topical ocular hypotensive medication delays or prevents the onset of primary open-angle glaucoma Arch Ophthalmol 2002 120 6 701 713 discussion 829–730 10.1001/archopht.120.6.701 12049574

[B41] Leske MC Heijl A Hussein M Bengtsson B Hyman L Komaroff E Factors for glaucoma progression and the effect of treatment: the early manifest glaucoma trial Arch Ophthalmol 2003 121 1 48 56 10.1001/archopht.121.1.48 12523884

[B42] Collaborative Normal-Tension Glaucoma Study Group The effectiveness of intraocular pressure reduction in the treatment of normal-tension glaucoma Am J Ophthalmol 1998 126 4 498 505 9780094 10.1016/s0002-9394(98)00272-4

[B43] Tsai JC Medication adherence in glaucoma: approaches for optimizing patient compliance Curr Opin Ophthalmol 2006 17 2 190 195 16552255 10.1097/01.icu.0000193078.47616.aa

[B44] Sridharan L Greenland P Editorial policies and publication bias: the importance of negative studies Arch Intern Med 2009 169 11 1022 1023 10.1001/archinternmed.2009.100 19506169

